# (-)-Epigallocatechin-3-gallate ameliorates learning and memory impairments by attenuating peroxidation in APP/PS1 transgenic mice

**DOI:** 10.1186/1750-1326-7-S1-S29

**Published:** 2012-02-07

**Authors:** Mingyan Liu, Fujun Chen, Tao Lin, Lutian Yao, Miao He, Zheng Zhu, Zhimin Yao, Shuang Wang, Weifan Yao, Zhenjie Zhang, Qiushi Tang, Minjie Wei

**Affiliations:** 1 Department of Pharmacology, School of Pharmaceutical Sciences, China Medical University, Shenyang, P. R. China

## Background

Alzheimer’s disease (AD), an age-related neurodegenerative disorder, is the predominant form of dementia in the elderly, clinically characterized by cognitive impairment and pathologically characterized by extracellular senile plaques largely composed of β-amyloid (Aβ) peptide. The overburden of β-amyloid (Aβ) deposition in AD is associated with peroxidation which plays an important role in neuronal dysfunction. APP/PS1 double transgenic mice, which display significant learning and memory impairments and the typical pathological changes such as numerous Aβ deposition, are ideal model to mimic the symptoms of AD. (-)-Epigallocatechin-3-gallate (EGCG) is the most abundant polyphenolic constituent in green tea, which has ironchelating, anti-inflammatory, antioxidant and anticancer activities. However, it remains unclear whether EGCG improves learning and memory impairments by attenuating peroxidation in APP/PS1 double transgenic mice. Therefore, we evaluated the relationships between the ameliorated memory dysfunctions and the inhibited peroxidative levels in APP/PS1 transgenic mice after EGCG treatment.

## Methods

APP/PS1 mice at the age of 9 months, randomly distributed into EGCG-treated APP/PS1 group (EGCG group) or APP/PS1 group, and age-matched wild-type mice (C57 BL/6J, WT group) were assigned as aging control. Mice were intragastrically administered EGCG (2 mg/kg) or vehicle (distilled water) once daily for 4 weeks. Memory function was evaluated in Morris Water Maze (MWM) and passive avoidance test (PAT). The contents of malondialdehyde (MDA), and the activities of total superoxide dismutase (t-SOD) and glutathione peroxidase (GSH-Px) in hippocampus were examined using lipid preoxidation MDA assay kit, total superoxide dismutase assay kit with WST-1, and total glutathione peroxidase assay kit, respectively. The protein expression of inducible nitric-oxide synthase (iNOS) in the hippocampus was measured by Western blot.

## Results

In PAT, the shorter latency and the increased error time to enter the dark compartment of APP/PS1 group were found compared with WT group (*P*<0.05). In MWM, the higher escape latency, longer path length and slower improvement in the navigation training for spatial acquisition, and the less passing frequency for mice to travel across the center of the removed platform and the less time spent in target quadrant in probe trial for memory consolidation, were dig out in these research (*P*<0.05), compared with WT group. After we found the learning and memory impairments of APP/PS1 mice, we evaluated the content of peroxidative substrate and the activities of antioxidative enzymes for further study. The increased contents of MDA, decreased activities of t-SOD and GSH-Px, and the overexpression of iNOS were detected, suggesting high levels of oxidative stress in the hippocampus of APP/PS1 mice. Moreover, EGCG treatment ameliorated the learning and memory deficits by improving the related parameters in MWM and PAT, compared with APP/PS1 group. And peroxidation was inhibited by decreased MDA contents, elevated activities of t-SOD and GSH-Px, and by downregulating the overexpression of iNOS in the hippocampus of APP/PS1 mice.

## Conclusions

These findings suggest that EGCG attenuates learning and memory impairment through ameliorating peroxidation in APP/PS1 transgenic mouse model, and may be a potential candidate as antioxidant agent for an AD medication.

